# Cognitive-Behavioral Therapy versus Other PTSD Psychotherapies as Treatment for Women Victims of War-Related Violence: A Systematic Review

**DOI:** 10.1100/2012/181847

**Published:** 2012-04-19

**Authors:** N. Inès Dossa, Marie Hatem

**Affiliations:** Département of Médecine Sociale et Préventive, Faculté de Médecine, Université de Montréal, Montréal, QC, Canada H3C 3J7

## Abstract

Although war-trauma victims are at a higher risk of developing PTSD, there is no consensus on the effective treatments for this condition among civilians who experienced war/conflict-related trauma. This paper assessed the effectiveness of the various forms of cognitive-behavioral therapy (CBT) at lowering PTSD and depression severity. All published and unpublished randomized controlled trials studying the effectiveness of CBT at reducing PTSD and/or depression severity in the population of interest were searched. Out of 738 trials identified, 33 analysed a form of CBTs effectiveness, and ten were included in the paper. The subgroup analysis shows that cognitive processing therapy (CPT), culturally adapted CPT, and narrative exposure therapy (NET) contribute to the reduction of PTSD and depression severity in the population of interest. The effect size was also significant at a level of 0.01 with the exception of the effect of NET on depression score. The test of subgroup differences was also significant, suggesting CPT is more effective than NET in our population of interest. CPT as well as its culturallyadapted form and NET seem effective in helping war/conflict traumatised civilians cope with their PTSD symptoms. However, more studies are required if one wishes to recommend one of these therapies above the other.

## 1. Introduction

### 1.1. Background

Warfare and torture occur on a large scale in many countries resulting in widespread death, disability, and trauma [1]. In 1997, Amnesty International reported that human rights violations had been recorded in over 150 countries worldwide [2]. Thus, the number of civilians affected by war, in comparison to military personnel, is rising because of the amount of cases of interpersonal violence [3]. According to the latest statistics of the United Nations High Commissioner for Refugees, there are approximately 42 million “forcibly displaced persons” worldwide of whom 15.2 million were refugees [4]. Those living in a war-affected area experience a range of severely traumatic experiences [5, 6]. For example, in the Democratic Republic of Congo, where a second civil war has been existent since 1998, it is common for civilians to be killed, kidnapped, sexually abused, enslaved, or tortured at gunpoint [7, 8]. Feeling that one's life is in danger, witnessing extreme violence or an individual's death, separation from ones family, or being detained against one's will, are diverse factors which can contribute to the development of various psychiatric diseases. This systematic review will focus on Posttraumatic Stress Disorder (PTSD), the most commonly studied consequence in relation to the atrocities previously cited.

PTSD was first recognized following the devastating war experiences of soldiers serving in Vietnam. Since then, the concept has been adequately applied in the assessment of various types of traumatic experience [9]. Most studies reported high prevalence rates of PTSD among tortured refugees (31% to 92%) and refugees affected by war trauma (23.5 to 77%) [9]. With the exception of 2 studies, those that examined gender differences in civilians' responses to war trauma found that females are more likely to develop PTSD than males [9].

Even in instances where only a few women are on the front line of combats, they and their children bear the brunt of its physical, socioeconomic, and emotional impacts [10]. In fact, because they are unarmed, weak and often unaccompanied during times of armed conflicts, adolescents and adult females are usually the first target of interpersonal violence. They are particularly vulnerable to sexual violence and intimidation at gunpoint, often used as a deliberate military and political tactic during armed conflicts [11]. It is common for combatants to use such action as a way of humiliating and attacking the community of the “enemy,” since women are viewed in many cultures as symbolic representatives of caste, ethnic, or national identity [7, 12]. Several reports provided by female victims of war mention that they are left to pick up the pieces of lives and societies shattered by gun violence [12]. Besides the infectious diseases (i.e., HIV), these victims often suffer from several gynecological problems and posttraumatic disorders, which can have a serious impact on reproductive health.

Physical and sexual violence committed against women during war time have always being condemned by institutions but more needs to be done to help the victims pick up the remaining pieces of life, regain confidence, and recover from their trauma. Unfortunately, there is limited and disparate information on what intervention is the most appropriate and effective for this category of victims. The primary objective of this systematic review is to assess whether the different forms of CBT can successfully help adult civilians (specifically women) who experienced war-or-conflict-related trauma (imprisonment, torture, sexual abuse, rape, kidnapping, or detainment against will) cope with the symptoms of PTSD and depression. Secondary, we identify which form of CBT is more efficient in reducing the severity of the previously cited outcomes.

### 1.2. PTSD and Different Forms of CBT

Given the clinical complexity of PTSD, it is not surprising that the development of treatments is quite challenging. Cognitive-behavioral therapy (CBT) includes a number of diverse but related techniques such as exposure therapy, stress inoculation training, cognitive processing therapy, cognitive therapy, relaxation training, dialectical behavior therapy and acceptance, and commitment therapy [13].

*Exposure therapy* (ET) refers to a series of procedures designed to help individuals confront thoughts, and safe or low-risk stimuli, that are feared or avoided [13]. Applied to the treatment of PTSD, most exposure therapy programs include imaginal exposure to the trauma memory and in vivo exposure to reminders of the trauma or triggers for trauma-related fear and avoidance [13]. In 2002, Neuner's research team developed a new form of ET called *Narrative Exposure Therapy* (NET). NET is a standardized short-term approach in which the classical form of ET is adapted to meet the needs of traumatized survivors of war and torture [14]. As most of the victims of organized violence have experienced many traumatic events, it is often impossible for them to identify the worst event before treatment. To overcome this difficulty, the team combined with ET, the testimony therapy's approach of Lira and Weinstein designed to treat traumatized survivors of the Pinochet regime in Chile [15]. Instead of defining a single event as a target in therapy, the patient constructs a narration of his whole life from birth up to the present situation while focusing on the detailed report of the traumatic experiences [14]. This treatment has shown low dropout rates in different studies. Many think that the main motivator of NET is the anticipation of receiving a written biography upon completion, that can help participants pass on their story to their children, while simultaneously educating them [16].

*Stress Inoculation Training *(SIT) is a multicomponent anxiety management treatment program that includes education, muscle relaxation training, breathing retraining, role playing, covert modeling, guided self-dialogue, and thought stopping [13]. *Cognitive therapy* (CT) predicated the idea that it is one's interpretation of an event rather than the event itself that determines emotional reactions. It involves identifying erroneous or unhelpful cognitions, evaluating the evidence for and against these cognitions, and considering whether the cognitions are the result of cognitive biases or errors, in the service of developing more realistic or useful cognitions [13]. *Cognitive processing therapy *(CPT) implements exposure to the trauma memory via writing a trauma narrative and repeatedly reading it, and is combined with CT focused on themes of safety, trust, power/control, esteem, and intimacy [13]. A culturallyadapted form of CPT was developed in 2004 by a team of researchers from Massachusetts to fit the needs of traumatised Cambodian refugees with pharmacotherapy-resistant PTSD [17]. *Dialectical behavior therapy* (DBT) is a comprehensive treatment developed for the treatment of individuals with borderline personality disorder. An important aspect of DBT is skills training in affect regulation and interpersonal regulation. Some trauma survivors may have deficits in these skill areas that render it difficult for them to tolerate or benefit from trauma-focused interventions such as ET [13].

CBT is the most studied treatments in the general population, and current guidelines recommend it as a first-line treatment for patients [13]. The different forms of CBT have been studied as treatment for PTSD since the early 1960s [18]. However, the amount and quality of evidence varies substantially from a program to another. CBT's different forms have been recognised as effective in helping survivors of trauma resulting from accidents, rape and crime witnessing; however, their effectiveness in helping war-or-conflict-related traumatised civilians is yet to be proven.

## 2. Methods

### 2.1. Search Methods for Identification of Studies

The recommendations of the Cochrane Handbook for Systematic Reviews of Interventions were followed in the design of the search strategy. The first author worked in collaboration with professionals at the Paramedical library of University of Montreal. Their role was to evaluate the research strategy and advise on the possible missed channels of search. The *Cochrane Central Register of Controlled Trials (CENTRAL)*, *CINAHL, EMBASE, Entrez-Pubmed*; *PsycInfo*, *Web of Science*, *MEDLINE (OvidSP),* and *USA clinical trials *databases were searched. In addition, the dissertation and thesis database was also reviewed for relevant publications. The keywords related to trauma (PTSD, posttraumatic stress disorders, stress disorders) were first used to extract publications on the subject. In order to refine the results to the population of interest (civilians, adults), studies conducted with the combatants (with keywords veterans and military personnel) and the young traumatised civilians (with keywords adolescent*, youth*, child*) were excluded. Since a lot of the interventions in countries affected by war/conflict are conducted by diverse humanitarian organisations (i.e., WHO, IANSA, UNHCR), these organisation websites were scanned for useful publications on the subject. A manual search was also performed using the reference list of reviews and books published on PTSD treatment in the population of interest [13, 19, 20]. This step was completed by searching potential publications with the keywords related to each form of CBT (exposure therapy, stress inoculation training, cognitive processing therapy, etc.).

### 2.2. Criteria for Considering Studies for This Paper

The inclusion criteria consisted of studies describing a randomised controlled trial in which the intervention compared any form of CBT to a control (no treatment, delayed treatment, treatment as usual, or non-CBT psychotherapy). The study needed to be designed to reduce PTSD symptoms among civilians who experienced one or multiple trauma during war-or-conflict. Study participants were required to be adults with the status of refugees, asylum seekers, or internally displaced. Trials conducted with participants traumatised by war/conflict related violence who were still living in their country of origin were also included. Studies with participants with comorbidities were accepted provided the primary objective of the trial was the reduction of PTSD symptoms. The main comorbid conditions found in the included studies were depression, anxiety, suicidal ideations, neck-focused and orthostasis-triggered panic attacks, with flashbacks during attacks and somatoform disorders. Most of the studies excluded participants with organic mental disorder, bipolar disorder, mental retardation, schizophrenia, psychosis, and drug abuse. The instruments used for the assessment of PTSD symptoms and severity had to be based on DSM's or ICD's criteria for a study to be eligible.

### 2.3. Data Collection and Analysis

The Cochrane Handbook for Systematic Reviews of interventions [21] was used to develop the methods of this paper. All studies resulting from search strategy were assessed for potential inclusion. They were excluded if the randomization process was incomplete, there was no control group or no data was reported. The studies in which civilians and army/police officers were combined were also excluded because of the different nature in which they are exposed to trauma. A data extraction form was designed. The first author performed the initial data extraction, and the Review Manager software (RevMan 5) was used to enter the data a second time. The articles' authors were contacted for further details on their studies if the trial conditions or data reported were unclear.

### 2.4. Assessment of Risk of Bias in Included Studies

The first author assessed the risk of bias in included studies considering criteria outlined in the Cochrane Handbook for Systematic Reviews of Interventions [22] and the second author reviewed the assessment for accuracy. Methods used for generation of the randomization sequence were described for each trial if reported. To assess the bias introduced into each study, five key domains were considered: sequence generation, allocation concealment, blinding of participants, blinding of assessors, and completeness of outcome data.

### 2.5. Missing Data

Data was analysed with the available information for each group participants according to their allocation.

### 2.6. Data Synthesis

A meta-analysis method with a random-effect was chosen since different instruments were used to assess PTSD or depression severity in the included trials. In fact, the use of a random-effects meta-analysis allows the incorporation of the heterogeneity existing among studies.

Two subgroup analyses were conducted with the Review Manager software (RevMan 5): the first assessed the effects of the treatments on PTSD score and the second assessed the effect of the treatments on depression score. The subgroup analyses also compared one form of CBT to the other. Each analysis investigates potential sources of heterogeneity, as differences in the type of intervention, participants' profile, or intervention setting (e.g., length and number of sessions) may affect the treatment effects. The analyses were conducted according to Deeks et al.'s method, integrated in RevMan 5 software [23].

## 3. Results

### 3.1. Results of the Search

**Figure 1:**
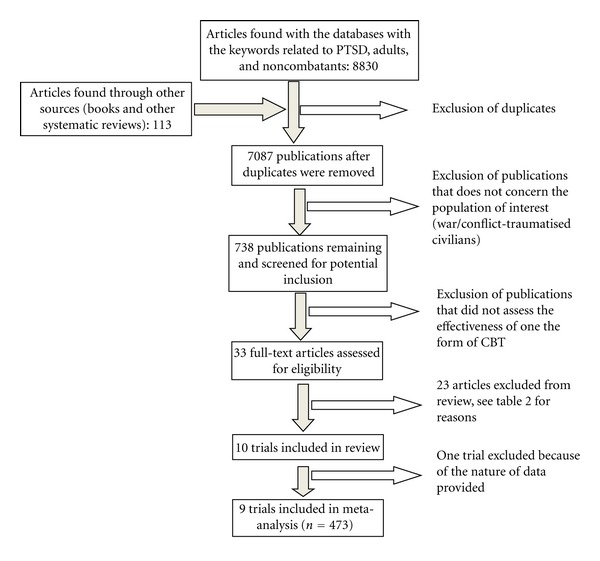
Flow chart of the systematic review.

Out of 738 trials initially identified from the databases, 33 publications studying the effectiveness of one of the forms of CBT were found.  Figure 1 presents the flow of information through the different phases of the review. None of those publications assessed the role of CT, ACT, or RT in PTSD symptoms reduction in the population of interest. Ten of the 33 trials were included in the paper. Tables 1 and 2, respectively, provide details of the included and excluded trials and in the last case the reason for their exclusion. 

 The two outcomes of interest in this paper (PTSD and depression severity) were monitored with different psychometric instruments and reported on a continuous scale (scores of PTSD and depression) (see Table 1 for details). Only three trials used an instrument that was adapted to their population of interest [17, 24, 25]. 

Among the ten studies included in this paper one assessed CPT's effectiveness in PTSD severity reduction [26], four studied culturallyadapted CPT's effectiveness [17, 24, 25, 27], and five assessed NET's effectiveness [16, 28–31]. The culturallyadapted CPT trials were conducted by a team of researchers located in Massachusetts (USA) with Cambodian and Vietnamese refugees [17, 24, 25, 27], who had been under pharmacotherapy for at least one year and were still meeting PTSD criteria. Even if the culturallyadapted CPT was their second treatment, its effectiveness could be measured since all patients continued use of their drugs and PTSD severity was measured at baseline. The five trials that used NET as the intervention group's treatment were conducted by German Researchers of University of Konstanz and Bielefeld [16, 28–31]. One trial conducted in Romania involved former political detainees [28], and three trials took place in Uganda with Rwandan and Somalian participants living in a refugee camp [16, 30, 31]. The fifth trial was conducted in Germany with asylumseekers from different origins [29].

Two out of ten trials required interpreters for the therapy sessions [16, 29]. Concerning the four culturallyadapted CPT trials, the therapy sessions were led by a therapist who was fluent in Cambodian. Finally regarding the three NET trials that did not require an interpreter, the therapy was either conducted by the therapists in the native language [28], or by lay counsellors and community-based lay therapists trained by the team of researchers [30, 31].

### 3.2. Risk of Bias in Included Studies

The quality of the included trials varied from one study to the other.  Table 3 summarizes each trial's quality according to the five criteria examined: adequate sequence generation, allocation concealment, incomplete outcome data addressed, and blinding of assessors, blinding of participants.

### 3.3. Effects of Interventions

Table 4 summarises the effects of each intervention on PTSD and depression scores. It also includes details about participant compliance to the allocated intervention and the PTSD remission rate in each group.

### 3.4. Subgroup Analysis: Culturallyadapted CPT versus CPT versus NET

We did not exclude the Neuner et al., 2008 [30] study which reported a high attrition rate because the risk of bias was judged as medium. In fact, the authors reported that dropouts did not significantly differ from treatment completers in age, nationality, number of event types, or pretest PTSD score and health scores (all *P*  values > 0.20). Furthermore, different circumstances of the refugee camps (independent of the treatment) could explain those dropouts which will then be random.

**Figure 2:**
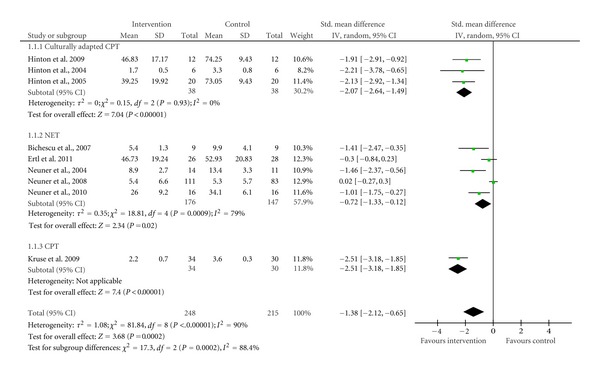
Forest plot of subgroup analysis culturallyadapted CPT versus NET versus CPT; Outcome: PTSD severity.

**Outcome 1: PTSD (Figure 2)**

One trial was excluded from the meta-analysis because rather than providing a global score, it provided the scores of severity for each criterion of PTSD symptoms (reexperiencing, avoidance/numbing, and hyperarousal).

The results indicate that culturallyadapted CPT can successfully help the adult civilians who experienced war-or-conflict related trauma. The effect size of 7.04 indicates that the intervention group (immediate treatment) outperformed the control group in terms of reduction of symptoms severity (delayed treatment) by seven times the standard deviation. This effect size is also highly significant at a level of 0.01 (*P* < 0.00001). One should note that there is no heterogeneity between the culturallyadapted CPT trials as indicated by the *I* square percentage (*I* ^2^ = 0%).

Figure 2 also shows that NET can successfully help adult civilians traumatised by war-or-conflict related violence to reduce the severity of their PTSD symptoms. The effect size of 2.34 is also significant at a level of 0.05 (*P* = 0.02). Contrary to the culturallyadapted CPT, there is a high heterogeneity between the NET trials (*I* ^2^ = 79%).

According to the results of our analysis, CPT compared to a control group (treatment as usual, which included social support, medical treatment as usual, and psychoeducation) can also successfully help reduce the severity of the PTSD symptoms in patients of our population of interest. The effect size of 7.40 was highly significant at a level of 0.01 (*P* < 0.00001).

In addition, a subgroup difference test was conducted. The results indicated that there is a significant difference between the effectiveness of the different forms of CBT versus a control (delayed treatment, treatment as usual, or psychoeducation/trauma counselling). After comparing the effect size, one can say that culturallyadapted CPT and CPT are more effective than NET in reducing the severity of PTSD symptoms.

**Figure 3:**
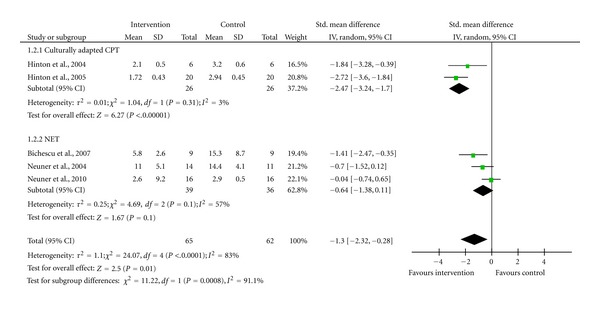
Forest plot of subgroup analysis culturallyadapted CPT versus NET versus CPT; Outcome: Depression severity.

**Outcome 2: Depression (Figure 3), Analysis Included Only the Five Trials Which Reported a Depression Score for Their Participants**

As indicated in Figure 3, culturallyadapted CPT can successfully help the population of interest reduce the severity of their depression symptoms. The effect size of 6.27 is highly significant at a level of 0.01 (*P* < 0.00001). Regarding the PTSD outcome, there is no heterogeneity between the trials (*I* ^2^ = 3%). The effectiveness of NET in the reduction of depression severity is low with an effect size of 1.67, which is not significant at a level of 0.01. However, the test of subgroup differences is significant at a level of 0.01 (*P* < 0.00008). This indicates that the effectiveness of culturallyadapted CPT in depression severity reduction is higher than NET's.

## 4. Discussion

This paper summarises the results of ten trials, which recruited a total of 473 men and women, all traumatised during war-or-conflict. The studies took place in different countries (industrialised and developing) characterised by a variety of health systems and existing protocol for psychological support offered to war- and conflict-traumatised individuals. Participants included Cambodian refugees resettled in USA [17, 24, 25], refugees from multiple countries in Germany [29], Somalian and Rwandan refugees [16, 30, 31], and Romanian tortured political detainees [28]. All the included studies were considered good quality because the absence of participants' blindness was not considered as important in this type of trial. In fact, this condition is difficult to meet because it is neither possible nor ethical to withhold from the patient information on the type of healthcare received.

The first subgroup analysis revealed that CPT, culturallyadapted CPT, and NET can significantly contribute to the PTSD severity reduction. However, the first two forms of CBT seem to have a higher effect on PTSD severity reduction than NET. We cannot exclude the possibility that those two forms of CBT might be the most adapted to the needs of patients of the population of interest. However, other reasons might have resulted in the superiority effect observed. First, it is possible that culturallyadapted CPT and CPT had a higher effect on PTSD severity reduction because they were all conducted in the participants' native language by people familiar with their culture. In fact, not all of the NET trials were conducted in the participants' native language, and the amount of trials available was too low to conduct a subgroup analysis that assesses the impact of interpreters on PTSD severity reduction. The observed difference might have also resulted from the variety of participants included in the NET trials compared to the other forms. In fact, the NET trials were conducted in different countries (Uganda, Germany, and Romania) with different settings and participants from different origins, while all the culturallyadapted CPT were conducted in Massachusetts with participants living in a more stable economic/politic context. It is possible that participants who are still living in the conflict-affected country are less inclined to fully benefit from the success of psychotherapy even when they need it. This idea derived from the comparison of the trial conducted by Bichescu et al. [28] to the ones conducted in Uganda [16, 30]. The former trial is the most effective, and this is likely due to the fact that the communist regime in Romania was over and most of the participants are living in a more peaceful environment than the refugees living in camps (most of them without decent lodging, food, or health services). Our hypothesis seems to be confirmed with the results of the trials that studied the effectiveness of NET among refugees in Germany, results which were in favour of the intervention.

The second subgroup analysis conducted revealed that culturallyadapted CPT and NET were effective in reducing the severity of depression in our population of interest. However, the effect size of NET therapy compared to control condition (Psychoeducation, Trauma Counselling or No treatment) was not significant. With regards to PTSD outcome, results show that culturallyadapted CPT is more effective than NET in depression severity reduction. The reasons described earlier might have also caused the results obtained.

A finding that is also important to discuss concerns the heterogeneity between the included trials. As reported earlier, there is a high homogeneity between the culturallyadapted CPT trials, results that can be explained by the fact that the three trials were led by the same therapist with similar participants in terms of type of trauma experienced (Cambodian genocide). In contradiction, a high heterogeneity between the NET trials was found, results that can be easily explained by the different settings of NET trials (several instruments used to assess PTSD severity, different number of sessions, variable length of session, interpreters recruited or not, participants from different origins, etc.).

CPT is the most studied treatment in relation to PTSD symptoms and severity in the general population. Moreover, it is the most recommended treatment in the general population based on the studies conducted in the industrialised countries. This form of CBT also seems efficient in helping war-traumatised adult civilians. In fact, when amalgamated with a meta-analysis, the CPT trials revealed a stronger effect size of the treatment compared to the control group (waiting list or delayed treatment). Our results also indicate that CPT seems the most indicated treatment to reduce depression severity. However, the trials' small sample sizes and the fact that every trial was conducted with patients originating from three countries (Bosnia for CPT, Vietnam and Cambodia for its culturallyadapted form) make it impossible to generalize CPT's effectiveness to every post war and conflict traumatised patient. Our results also emphasize the necessity of taking culture into account while designing interventions for PTSD patients or refugees of non-western countries since the culturallyadapted CPT seems more effective than the NET in PTSD and depression severity reduction.

In comparison to CPT, NET is a more contemporary method and more researched method of treatment in the population of interest (5 out of 10 trials compared it to another type of therapy). NET also seems to be the most adapted method to our population of interest because it is designed to suit their need and its effectiveness has been proven in different trials. Our meta-analysis also confirmed that it can help subjects of our population of interest in lowering the severity of their PTSD and depression symptoms. Even if NET does not appear to be the most effective treatment, the fact that it has been applied with success to participants from diverse origins (Romania, Somalia, Sudan, Turkey, Balkans, and Uganda) makes it easier to generalise its effectiveness among the population of interest. We also think that this form of CBT will probably benefit from integrating the variable of culture into the design of their sessions.

As indicated in the title of this paper, we were interested in assessing the effectiveness of the different forms of CBT according to the sex of the participants in order to find which treatment is the most effective for women. Unfortunately, this was not possible because even if most of the trials included men, and women none of them reported their results according to the sex or gender of participants. We think that subsequent trials should assess the difference of treatment effect between men and women since previous studies on PTSD symptoms and its severity revealed a difference of its rate between men and women in the same population, women being at higher risk of developing it [32–34]. Thus, we highly suggest that future researches take the sex/gender variable into account since an increasing number of studies suggest its role in several health matters [35, 36].

## 5. Potential Biases in the Review Process

First, the selection of a specific category of war-traumatised patients (adults) might seem arbitrary. We thought that it is important to separate adults from young ones because the type of CBT and NET treatment used in those categories are not the same. We also separated those categories of age because we felt that the process of healing might be different between an adult who is usually the victim of violence and a child/adolescent who is usually a witness and/or victim of that violence. Secondly, although an extensive list of databases was used for study selection, we cannot rule out the possibility that some articles may have been missed with our research strategy. In fact, since five out of ten of the studies were conducted by German researchers we cannot exclude the fact that some of their studies were not found in the English databases used.

## 6. Main Conclusions of the Study and Implications for Practice

This systematic review demonstrates that culturallyadapted CPT, CPT, and NET can successfully help war-or-conflict-traumatised civilians in reducing their PTSD symptoms. However, only the culturallyadapted CPT seems effective in reducing the depression score of civilians who experienced war-or-conflict related violence. Even if the subgroup analysis clearly demonstrates that CPT and its culturallyadapted form are more effective than NET, more evidence is needed in order to specifically recommend one of the forms of CBT over the other for our population of interest.

## 7. Implications for Research

### 7.1. Recommendations for Study Designs

More research needs to be carried out on CBT (specifically its culturallyadapted form) and NET treatment effectiveness in PTSD severity reduction within our population of interest. In fact, these people need to cope with the symptoms, in order to be able to contribute to the prosperity and well-being of their societies. It is also important that researchers agree on common characteristics which will allow conduct of a better comparison between studies in the future, in order to recommend a specific treatment. They should agree on the instruments, the number of sessions given, and their length. For example, in the CBT trials the number of sessions was different from one study to another. It is also important that the same type of control group be used for each trial (delayed-treatment or treatment as usual). In fact, there was a difference in the type of control group between the trials that used NET as an intervention. Trials comparing these two methods to a standard treatment might also be a good idea and collaboration between the varying teams (Hinton and Neuner teams) would certainly help this. To ensure the quality of the trials, researchers must also be sure to conceal allocation (none of the reviewed studies did so) and blind the assessors (followed by a test of the blindness integrity). Moreover, researchers must make certain that enough participants in the intervention and control group are included in the trial. In fact, a key limitation in most studies is the sample size. Only one study included an adequate number of participants (*n* = 111) in the intervention group, but also reported a high number of losses to followup, which made the results partly subject to the risk of bias such as incomplete outcome data.

### 7.2. Integrating Cultural Validity

There is not only a question of cultural validity regarding the PTSD itself (and its associated comorbidities), but also the instruments and specialists used to assess it remain important. In fact, some argue that PTSD symptoms may have various values or meanings depending on the different cultures, and some symptoms may not be perceived as distressing among certain groups [37]. The validity of applying a western-based trauma model, and the label PTSD to people from nonwestern countries has also been questioned [38]. Summerfield has even raised some fierce criticisms on the programs that do not integrate the question of validity when working with nonwestern populations. Surprisingly, none of the NET trials included lead researchers originating from the refugee population, a situation that most likely reduces the possibility of including the aspect of cultural validity in the intervention.

### 7.3. Research on Other Promising Treatments

Aside from NET and CPT, different therapies have been studied in our population of interest. One of them, *testimonial psychotherapy*, was used as treatment for the intervention group in an RCT [39]. This intervention was conducted with nondisplaced Mozambican civilians who had experienced trauma during a civil war in Mozambique. Several writers have noted that testimony psychotherapy offers survivors some therapeutic benefits in their trauma recovery [40]. As NET, it allows participants to have a written testimony at the end of the treatment with a benefit of a high completion rate. *Family group therapy* has also been described as potentially efficient in the population of interest. This treatment might be even more effective when several patients or traumatized persons belong to the same family, a situation that is fairly common [41–43]. This therapy gives the patients the advantage to receive social support from their family which facilitates their access and use of mental health services. Unfortunately, none of the interventions using this method were in the form of an RCT.

## Figures and Tables

**Table 1:** Participants, measures, characteristics of interventions, and assessments' timing in the included trials.

Studies (author, year)	Form of CBT offered (number of sessions and length)	Description of trauma	Participants and repartition by sex	PTSD diagnosis and severity assessment	Depression assessment	Number of assessments and timing
Hinton et al., 2009 [27]	*Culturally Adapted CPT, immediate versus delayed treatment. * Participants received 12 weekly sessions of adapted CPT, (duration not reported)	Type of trauma not specified	24 Cambodians with a pharmacology-resistant PTSD. Participants passed through Cambodian genocide and were at least 6 years old at the beginning of the genocide. 60% of participants were women	PTSD severity assessed with Clinician Administered PTSD Scale or CAPS (CAPS; Weather et al., 2001) [44] validated within the Cambodian population.	None	3 assessments for the immediate-treatment group and 2 for the delayed-treatment group.

Hinton et al. 2004 [17]	*Culturally Adapted CPT versus control (delayed treatment) * Each group followed 11 sessions (duration not reported)	Types of trauma not specified.	12 Vietnamese participants affected to either Immediate treatment (IT) group or Delayed-treatment (DT) group. Women represent 50% of participants.	PTSD diagnosed with Structured Clinical Interview for DSM-IV (SCID; First et al., 1995) [45]. PTSD severity assessed with the Havard Trauma Questionnaire (HTQ) translated and validated for the Vietnamese population (Mollica et al., 1992) [46].	Hopkins Symptom CheckList-25 (HSCL-25)	3: at pretreatment, after IT finished sessions of CPT and after DT had undergone CPT.

Hinton et al. 2005 [24]	*Culturally Adapted CBT versus control (delayed treatment) * Each group followed 12 weekly sessions (duration not reported)	Types of trauma not specified.	40 Cambodian participants (survivors of the 1975–1979 Cambodian genocide) affected to either IT or DT groups. Women represent 60% of participants of each group.	PTSD diagnosed with SCID. PTSD severity assessed with CAPS validated within the Cambodian population.	Symptom Checklist-90-R's depression subscale.	4: at pretreatment, after IT finished CPT, after DT finished CPT and at 3 months posttreatment for both groups (followup).

Otto et al. 2003 [25]	*Culturally Adapted CPT versus control (sertraline alone) * Ten sessions (duration not reported) of CBT were delivered to the intervention group.	Types of trauma not specified.	10 participants were allocated to pharmacotherapy alone or pharmacotherapy + psychotherapy. All participants were women.	PTSD diagnosed with SCID (First et al., 1995) [45]. PTSD severity was assessed with CAPS. PTSD subscales (re-experiencing, avoidance and arousal) were examined as separate outcome variables.	HSCL-25 validated for the Khmer population.	One: posttreatment

Bischescu et al., 2007 [28]	*NET versus PED * NET group received 4 weekly or biweekly sessions of 120 minutes each. Treatments were performed within a time period of 10 weeks.	Trauma was experienced during imprisonment. Types were not specified.	18 participants (former political detainees) were allocated to either NET or PED. Distribution of participants' sex was not reported.	Composite International Diagnostic Interview (CIDI; WHO, 1997) [47]. Patients were also asked to describe their specific symptoms or give examples during assessments.	*Beck Depression scale* (Beck, 1978; Beck and Steer, 1987) [48, 49].	2: before and after treatment (six months postintervention)

Neuner et al., 2010 [29]	*NET versus Treatment As Usual*. Patients of the NET group followed between 5 and 17 sessions (mean = 9 and sd = 3.77) of approximately 120 mins. The 2 groups received one PED session at trial beginning.	Witnessing a violent assault on a familiar person, torture, being in a war zone, and experiencing a violent assault by a stranger.	32 Asylum seekers with a history of victimisation by organised violence allocated to either NET or treatment as usual (TAU) representing the control. Women represent 31.2% of participants.	Posttraumatic Diagnostic Scale (PDS; Foa et al., 1995) [50].	HSCL-25.	2: pre and post treatment

Neuner et al., 2004 [16]	*NET versus PED (no treatment) * *NET versus SC * PED group had 1 session of treatment. NET and SC groups had 4 sessions of therapy. Sessions lasted 90 mins, exceptionally 120 mins.	Witnessing people badly injured or killed; threats with weapons, kidnappings, attacks, torture, combat experiences, sexual assaults and natural disasters.	43 participants allocated to either NET or SC interventions with PED as control. Women represent, respectively, 75%, 57.1% and 53.3% of PED, SC and NET groups.	CIDI *(CIDI; WHO, 1997) *[47]* and* Posttraumatic Diagnostic Scale (PDS; Foa et al., 1995) [50].	Self-reporting Questionnaire-20 (SRQ-20; Harding et al., 1980) [51].	4: pre-treatment, post-treatment, 4 months and 1 year after treatment.

Neuner et al., 2008 [30]	*NET versus no treatment (MG) * *NET versus TC * Participants received six sessions (lasting between 1 and 2 hrs) of NET or TC.	Number of traumatic events was reported, but types of trauma were not.	277 participants (Rwandan and Somalian refugees) were allocated to either NET, TC (Trauma counselling), or MG (monitoring group). Women represent, respectively, 49.1%, 53.2% and 50.5% of MG, TC, and NET groups.	CIDI and PDS.	None	3 times for NET and TC groups: at pre-treatment, 3 and 6 months posttreatment. MG was tested at pretreatment, 6 and 9 months.

Ertl et al. 2011 [31]	*NET versus Academic catch-up or waiting list. * Participants received 8 sessions of NET.	Abduction/ experienced or witnessed trauma.	85 formerly abducted youths were allocated to one the 3 groups. Between 42 and 67% of the participants of each group were women.	CAPS.	MINI.	4 assessments for each group at pretreatment, 3, 6 and 12 months.

Kruse et al. 2009 [26]	*CPT versus usual care. * 25 hours of manualized trauma-focused psychotherapy (CPT)	Torture, mass rape, genocide, expulsion	Participants (Bosnian) were between 18 and 61 years old and without no serious illness or alcohol/drug dependence. 67.7% of participants were women.	Havard Trauma Questionnaire (PTSD event section); Symptom Checklist (SCL-90R)	None	2 assessments: before and after intervention.

**Table 2:** Trials excluded from this paper and reason for exclusion.

Study	Authors, year	Form of CBT studied	Reason for exclusion
[52]	D'Ardenne et al., 2007	CPT	Three intervention groups and no control. Randomization process was not applied.

[53]	Duffy et al., 2007	CT	27% of intervention group and 28% of delayed treatment group were police or army officer.

[54]	Grey and Young 2008	CPT	A case study.

[55]	Hinton and Otto 2006	Somatically-focused CPT	Describes only the benefit of considering a somatic-focused CBT.

[56]	Schulz et al., 2006	CPT	Not a randomized controlled trial.

[57]	Stenmark et al., 2008	NET versus Usual care	Recruitment and interventions ongoing at time of review.

[58]	Heilmann and Måkestad 2008	NET	Some participants have not experienced war-or-conflict related trauma. Absence of data on control group.

[59]	Jacob et al. submitted	NET	Not yet completed by authors; data not available.

[60]	Halvorsen and Stenmark 2010	NET	No randomization, only one intervention group was assessed before and after therapy sessions.

[61]	Flaxman and Bond 2010	SIT versus ACT	Participants were not war/conflict-traumatized civilians.

[62]	Iverson et al., 2011	CPT	Participants were not war/conflict-traumatized civilians.

[63]	Galovski et al., 2009	CPT	Participants were not war/conflict-traumatized civilians.

[64]	Otto and Hinton 2006	Modified ET	No quantitative data reported.

[65]	Paunovic and Öst 2001	CPT versus ET	No control group. The two groups received a form of CBT.

[66]	Hensel-Dittmann et al., submitted	NET versus SIT	No control group. The two groups received a form of CBT.

[67]	Wagner et al., 2007	DBT	No quantitative data but only qualitative description.

[68]	Somnier and Genefke 1986	Not indicated	No quantitative data. Type of therapy unclear.

[69]	Tarrier et al., 1999	CT versus ET	Patients did not experience war trauma and the two groups received a form of CBT (no control group).

[70]	Tarrier et al., 1999	CT versus ET	Patients did not experience war trauma and the two groups received a form of CBT (no control group).

[71]	Boehlein et al., 2004	Not indicated	Not a randomized controlled trial.

[14]	Neuner et al., 2002	NET	A case report.

[72]	Schulz et al., 2006	CPT	Not a randomized controlled trial.

**Table 3:** Risk-of-bias table of the 9 included trials.

Study	Adequate sequence generation	Allocation concealment	Incomplete outcome data addressed	Blinding of assessors	Blinding of participants
Bichescu et al., 2007 [28]	Yes: assignment through a random selection procedure (name-cards) to either NET or PED group	No	Yes: no dropout reported among participants who started trial	No: an attempt was made. Blinding was finally impossible due to the large differences in procedures and number of sessions between the 2 groups	No: an attempt was made. Blinding was finally impossible due to the large differences between the two groups

Hinton et al., 2004 [17]	Yes: participants were all randomly assigned but the method was not described	No	Yes: no dropout reported among participants who started trial	No: no attempt was made	No: no attempt was made

Hinton et al., 2005 [24]	Yes: patients were stratified by gender with random allocation to either the IT or DT group decided by a coin toss	No	Yes: no dropout reported among participants who started trial	Yes: but blinding's integrity was not tested	No: no attempt was made

Neuner et al., 2004 [16]	Yes: patients were randomly assigned to either NET, SC or PED group by using a dice	No	Yes: missing data were estimated with a restricted maximum likelihood procedure.	Yes: interviewers were blinded for participant's treatment condition.	No: no attempt was made

Neuner et al., 2008 [30]	Yes: patients were randomly allocated to a group by altering allocation of randomly ordered participants. However, method was not described	No	Yes: but partly. Authors reported a high global attrition rate, 23%, 53.1% and 61% at, respectively, 3 months, 6 months and 9 months. Authors chose to apply mixed-effects models instead of a last-observation-carried-forward (LOCF) procedure, considered too conservative.	Yes	No: no attempt was made

Neuner et al., 2010 [29]	Yes: participants were randomized to NET or TAU group with a block permutation procedure with blocks of 4 patients	No	Yes: low dropout rate (6.3%). Authors used mixed effects models instead of an LOCF procedure to handle missing data. This method did not probably introduce a significant bias because of the small number of drop outs (2)	No: blindness could not be maintained in all cases so we cannot rule out an assessor bias	No: no attempt was made

Otto et al., 2003 [25]	Yes: participants were randomly assigned to either Sertraline alone or Sertraline + CBT, but method was not described	No	Yes: no dropout was reported during trial	No: no attempt of blinding assessors was made	No: no attempt was made

Ertl et al., 2011 [31]	Yes	No	Yes: mixed effects model was used	Yes: psychologists were blinded to treatment conditions	No

Hinton et al., 2009 [27]	Yes: random allocation by a coin toss	No	Yes	No	No

Kruse et al., 2009 [26]	Yes: first 35 patients assigned to intervention group	No	Yes	No	No

**Table 4:** Effectiveness of the different forms of CBT, compliance rate to allocated intervention & remission rate in each group.

Trial	Form of CBT	Effectiveness of the therapy on PTSD/depression severity	Compliance rate to allocated intervention	Remission rate in groups
Hinton et al., 2009 [27]	*Culturally Adapted CPT, immediate versus delayed treatment*	*PTSD*: between-group effect size based on first and second assessment = 1.98. Depression: Effect not reported	100% in immediate and delayed treatment groups	Not reported

Hinton et al. 2004 [17]	*Culturally Adapted CPT versus control (delayed treatment)*	*PTSD*: between-group effect size based on first and second assessment = 2.5 *Depression:* Between-group effect size based on first and second assessment = 2.0	100%	Not reported

Hinton et al. 2005 [24]	*Culturally Adapted-CBT versus control (delayed treatment)*	*PTSD*: between-group effect size based on first and second assessment = 2.17 *Depression*: Between-group effect size based on first and second assessment = 2.77	100%	Not reported

Otto et al. 2003 [25]	*Culturally Adapted CPT versus control (sertraline alone)*	*PTSD*: global score not provided. Effect size for re-experiencing, avoidance/numbing and hyperarousal were, respectively, 0.82, 0.85 and 0.45 *Depression*: Between-group effect size = 0.	100%. 40% of patients in each group reported at least one, mild, adverse symptom with treatment. The most common adverse effects were fatigue and nausea, and none resulted in treatment discontinuation.	Not reported

Bischescu et al., 2007 [28]	NET versus PED	*PTSD*: effect size between-groups = 3.15 *Depression*: Between-group effect size = 0.97	100%	At 6 months post-treatment, 5 out of 9 (56%) of NET group participants were PTSD free while only 1 out of 9 (11%) patients of the Psychoeducation group was in remission.

Neuner et al., 2010 [29]	*NET versus No Treatment (TAU)*	*PTSD*: effect size between-groups = 1.6 with *Depression*: Between-group effect = 0.8	87.5%	Not reported.

Neuner et al., 2004 [16]	*NET versus PED (no treatment)* *NET versus SC *	*PTSD*: effect size between-groups = 1.9 *Depression*: Between-group effect size = 1.1	100%	At 1 year followup, 29% of the NET group (4 participants), 79% of the Supportive counselling group (11 participants) and 80% of the Psychoeducation group (8 participants) were still PTSD positive

Neuner et al., 2008 [30]	*NET versus no treatment (MG)* *NET versus TC*	*PTSD*: effect size between-groups = 1.4 *Depression*: not reported	96.4% for NET group	At 9 months followup, 69.8% of the NET group (30/43), 65.25% of the Trauma Counseling group (30/46) and only 36.8% of the control group (7/19) no longer fulfilled the criteria for PTSD.

Ertl et al. 2011 [31]	*NET versus Academic catch-up or waiting list.*	*PTSD*: effect size between NET and waiting list = 1.8 *Depression:* Between-group NET and waiting list = 0.38	85.7% for NET group	Not reported

Kruse et al. 2009 [26]	*CPT versus usual care*	*PTSD*: effect size between-groups = 2.7 *Depression*: Not reported	97% for CPT group	Not reported
